# The organotin contaminants in food: Sources and methods for detection: A systematic review and meta-analysis

**DOI:** 10.1016/j.fochx.2021.100154

**Published:** 2021-11-05

**Authors:** Parisa Sadighara, Mahdi Jahanbakhsh, Zahra Nazari, Parisa Mostashari

**Affiliations:** a Department of Environmental Health, Food Safety Division, Faculty of Public Health, Tehran University of Medical Sciences, Tehran, Iran; b Food Quality and Safety Research Group, Food Science and Technology Research Institute, ACECR Mashhad Branch, Mashhad, Iran; c Department of Food Science and Technology, National Nutrition and Food Technology Research Institute, Faculty of Nutrition Sciences and Food Technology, Shahid Beheshti University of Medical Sciences, Tehran, Iran; d Nutrition and Food Sciences Research Center, Tehran Medical Sciences, Islamic Azad University, Tehran, Iran

**Keywords:** Organotin, Contamination, Food, Detection, Systematic review

## Abstract

Organotin compounds in low doses have toxic effects. These components may contaminate food. The aim of this systematic review was to determine the type and level of organotin in food that are mainly contaminated with these compounds, as well as common detection methods. The research studies with keywords Organotin, Tributyltin, TBT, Food, Detection, Contamination, and Pollution were searched in PubMed, Scopus, Science Direct, and Google Scholar databases, regardless of publication time. Two author independently investigated the publications. A number of 123 studies were obtained and only 9 articles were finally selected according to exclusion and inclusion criteria. Studies were selected which organotin components were detected in the food matrix. The important data were extracted. Meta-analysis was calculated for the amount of TBT in seafood. The most important of these compounds are TBT, TPhT, Dibutyltin (DBT) and di-*n*-octyltin (DOT). Surveys were conducted on three continents, Europe, America and Asia. Contaminated foods reportedly included seafood and edible oils, according to studies. TBT was investigated more than other tin components in food. The overall average estimate for TBT in seafood was estimated at 182.33 ng/g that This amount was more than maximum limit. Therefore, it is necessary to take measures to treat the wastewater so that these harmful compounds do not reach the water of sea.

## Highlights

• This was the first systematic review concerning organotin in food.
• Among the various organotin, TBT was reported more than others.
• The overall mean of TBT in seafoods more than permissible maximum level.

## Introduction

Organotin has a variety of uses, including stabilizers in plastics, antifoam in paints, wood preservative and pesticide(Chen et al., 2019, He et al., 2020). 40% of organotin compounds are used in plastics(Liu & Jiang, 2002). These components are recognized as organometallic components(Zhu et al. 2013). The organotin have been identified in human blood and liver samples(Forsyth & Casey, 2003). European food safety authority(EFSA) set a tolerable daily intake (TDI)of 0.25 µg/kg for the four organotin. These four compounds include Tributyltin(TBT), Triphenyltin (TPhT), DBT and di-*n*-octyltin (DOT) (Chung et al. 2020). The origin of these compounds is mainly due to human activities(Sousa et al., 2009, Zabaljauregui et al., 2007). TBT and TPhT have the most toxic effects on the endocrine glands and are banned in many countries(Yang et al. 2010). EU and US regulations prohibit the use of TBT and TPhT in food contact materials(He et al. 2020). TPhT is used as a fungicide in agriculture(Forsyth et al. 1992). Dibutyltins (DBTs) and TBT are neurotoxic and damage the bile duct(Amodio-Cocchieri et al. 2000). Tributyltin has more toxic effects (Amodio-Cocchieri et al., 2000, Vacchina et al., 2020). Tributyltins have toxic effects on aquatic populations even at very low doses(Amodio-Cocchieri et al. 2000). This kind of organotin has genotoxic effects(Santos et al. 2009). They lead to deformity of crab limb and death of mussel larvae(Forsyth & Casey, 2003). TBT oxide also induce mutations and have teratogenic(Amodio-Cocchieri et al. 2000). TBT has been identified in humans as an immunotoxic compound, and the tolerance daily intake was calculated 0.25 mg/kg body weight/ day based on immunological studies(Forsyth & Casey, 2003). The tolerance daily intake is 250 ng/ kg per body weight for total of four organotin TBT, TPT, DOT, and DBT(Rantakokko et al. 2006). These compounds are also persistence in the environment(Sousa et al. 2009). These compounds are also found in house dust. According to studies conducted in house dust, monobutyltin (MBT) , another organotin is found more than other compounds in house dust(Kannan et al. 2010). This dust may be swallowed by children, so they will be more exposed to tin compounds than adults(Kannan et al. 2010). Furthermore, in the blood test of working women of reproductive age, MBT level was higher than other organotin(Program 2016). This type of organotin is used as a dose reference(Sousa et al. 2017).

The organotin in food are absorbed by cells in the gastrointestinal tract and enter the bloodstream(Sant’Anna et al. 2012). In order to know that the contaminants, toxins and chemicals in food are in the tolerance daily intake (TDI) range, it is necessary to know the level of these substances in food. Therefore, the objective of this systematic review was to determine the amount and type organotin component in food.

## Methods

This systematic review was written on PRISMA checklist. Two authors performed all stage including inclusion and exclusion criteria, and data extraction to prevent bias.

### Search strategy

The articles in English language were searched on 16 June 2021. There was no time limitation. The chosen databases were PubMed, Science direct, Google scholar, and Scopus. The keywords for searching were set: (Organotin OR Tributyltin OR TBT) AND (Food) AND (Detection) AND (Contamination OR Pollution). A total number of 123 articles were identified from databases. At first, the title and abstract of the manuscript were reviewed. Manuscript that did not meet the inclusion criteria were excluded from the study. The full text were assessed with two author (P.S and Z.N).Then, the full text of the selected Manuscript was carefully studied and the data was extracted according to the protocol.

### Inclusion and exclusion criteria

The two reviewers (P.S and M.J) searched the keywords in databases independently. Invitro and animal study, environmental sample, review and chapter of book, non-English article, biomonitoring, and application of organotin were excluded. Inclusion criteria for this systematic review included original articles that measured organotin levels by valid methods of measuring. All publication that was according to inclusion criteria was assessed. The sample size of a significant number of studies was only one, so it was excluded from this systematic review.

### Data extraction

The name of the first author, time of study, country, type of food and organotin, amount and sample size, method of measuring in samples were extracted in the Table 1. The data extracted by two reviews (P.S and M.J) indecently. In all steps, disagreement were consulted with third author. If the full text of the articles was not available, the authors of the article were emailed.

**Table 1:** The type of food organotin and the detection method according to the published data.

Analysis method/unit of detection	Quantity	Type of food /sample size	Type of organotin	Country	Authors / Year
GC-PFPD µg/g	0.008 to 0.135	Bivalves N = 5	Tributyltin(TBT)	Japan	Inoue/2006 (Inoue et al. 2006)
GC-PFPD µg/Kg	TBT:Farm fish 2 (1– 21) TBT:Free living fish 5 (1– 86)	Farm fish = 66 Free living fish = 49	TBT DBT	Italy	Amodio-Cocchieri/2000 (Amodio-Cocchieri et al. 2000)
	DBT:Farm fish 4 (1– 28) DBT: Free living fish 4 (1–71)				
GC-PFPD ngSn/g	Tributyltin = 602.3 ± 14.5 dibutyltin = 368.7 ± 5.5 monobutyltin = 203.7 ± 4.0	an edible gastropod N = 2	TBT DBT MBT	Chile	Mattos/2017 (Mattos et al. 2017)
GC–MS µg/g	TMT = 13.86 ± 0.31 DMT = 1700 ± 40.0 MMT = 225.06 ± 7.5	lard samples sample size = Not mentioned	Trimethyltin chloride(TMT) Dimethyltin dichloride(DMT) Monomethyltin trichloride(MMT)	Chine	Gui-bin/2000 (Gui-bin &Qun-fang 2000)
GC-ICP/MS µg/Kg	Mean concentration of TBT = 0.32 Mean concentration of DBT = 0.04 Mean concentration of DOT = 0.01 Mean concentration of TPhT = 23	Fish N = 201	TBT DBT DOT TPhT	Hong Kong	Chung/2020 (Chung et al. 2020)
	Mean concentration of TBT = 0.19 Mean concentration of DBT = 0.31 Mean concentration of DOT = 0.02 Mean concentration of TPhT = 15	crustaceans and molluscs N = 109			
GC–MS ngSn/g	The maximum amount of: MBT 24.2 ± 1.3 DBT 46.0 ± 0.7 TBT 68.1 ± 20.1 MPhT 589 ± 25.6 DPhT 992 ± 18.9 TPhT 747 ± 7.3	commercial oysters N = 20 for six seafood markets	MBT,DBT,TBT, MPhT ,DPhT TPhT	Chine	Chen/2019 (Chen et al. 2019)
HPLC–MS/MS µg/kg	DPhT and TPhT was ND in all oil samples. The maximum amount of TBT in blended oil was 28.8 ± 2.82, followed by Sunflower seed oil 26.9 ± 2.15 and Soybean oil 13.1 ± 1.24 DBT was measured in all oil samples except sesame and rape oil. The maximum amount of DBT in blended oil was 12.3 ± 1.09	edible vegetable oil sample size of each oil = 5	TBT DBT DOT TPhT	China	Liu/2016 (Liu et al. 2016)
GC-PFPD µg/g	All organtin compounds were ND in all fish species except two: 1)TPT in Mediterranean codling 3.5 ± 0.8 2)DBT in Gunther grenadier4.9 ± 4.3	Common mora Mediterranean codling Gunther grenadier Risso smooth-head Spiderfish N = 3 for each of species	MBT, DBT, TBT, MPhT, DPhT and TPhT	Spain	B o r g h i/2002
GC-PFPD µg/g	DBT in Tuna:0.18 ± 0.25, Shrimp:0.14 ± 0.08, Oyster:0.024 ± 0.025 TBT in Tuna:ND, Shrimp:ND Oyster:0.056 ± 0.032 TPT in Tuna:ND, Shrimp:0.010 ± 0.013 Oyster:ND DPT in all of sample:ND	fish and shellfish N = 5	DBT, TBT, DPT,TPT	Japan	Tsunoda/1993 (Tsunoda 1993)

### Estimation of the mean of tributyltin in seafood

Among the organotin, tributyltins was reported more than others, so this compound was selected for *meta*-analysis. The levels of tributyltins in seafood were converted to ng/g units. For this estimation, studies were selected that had mean, standard deviation and number of samples. The total mean was estimated with comprehensive *meta*-analysis software.

## Results

### The search processes

**Fig. 1:**
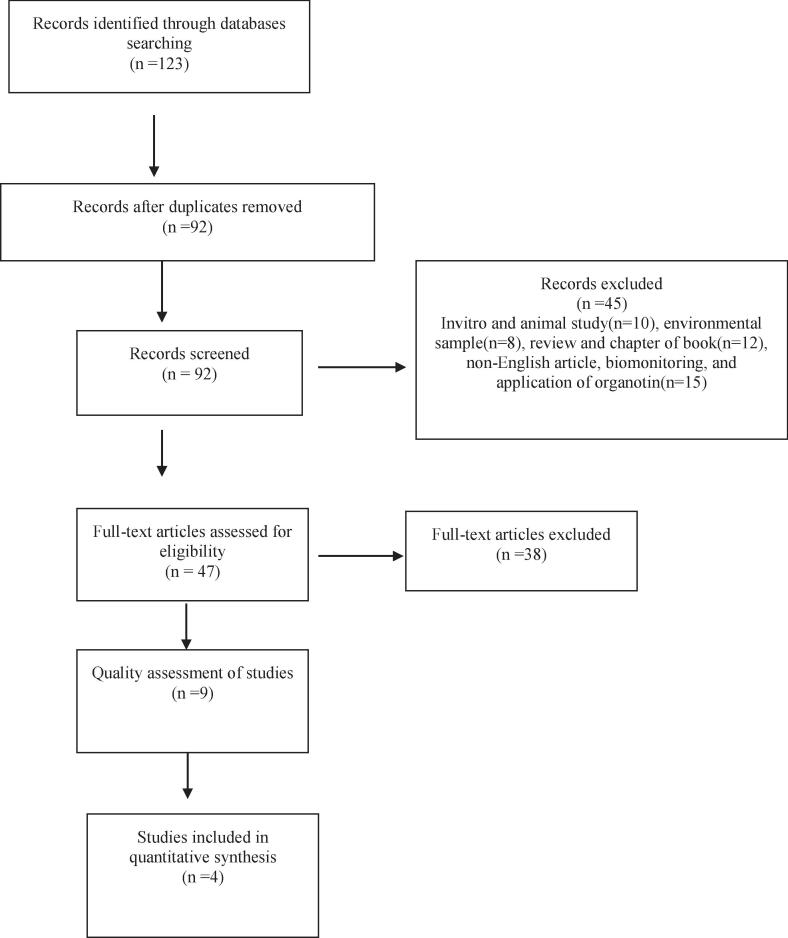
The diagram of study.

123 articles were achieved by searching in PubMed, Scopus, Science direct, and Google Scholar database. 31 articles were excluded from the study due to duplicating. The title and abstracts of the remaining articles were carefully studied and 45 articles were excluded because of using review and chapter of book, animal studies, other sample including environmental sample, application and non-English article. Then, the full texts of 47 papers were taken. The quality of the studies was also assessed by two persons. At end step, 9 articles were chosen. The PRISMA checklist was conducted for this systematic review. Fig. 1 shows PRISMA diagram of database searches.

### The descriptive results of screened manuscript

Of all screened manuscript, 9 were selected for the systematic review. The type of food and type of organotin identified and the method used to identify organotin are shown in Table 1. Important results of each study are listed in the discussion section.

### Estimation of the mean of tributyltin in seafood

Four of the studies in the table had the mean, standard division and sample of size for TBT in seafood. Therefore, 4 publications were selected in the present study for *meta*-analysis. The overall mean was estimated with the random model. This was estimated at 182.33 ± 84.62 ng/g.

## Discussion

In this study, tin compounds were observed in seafood and liquid oils. The extracted data shows that most of the samples examined are foods of marine origin. According to previous studies, tin compounds are a global threat to marine ecosystems(Hu et al. 2006). TBT, DBT and MBT have been stable in marine ecosystems for many years. In a study, the exposure to all organotin was investigated, it was observed that most exposure occurs through fish and crustaceans(Rantakokko et al. 2006).This indicates the contamination of food of marine origin from the water and the entry of these compounds from the packaging. Waters are likely to contain a variety of contaminants, including heavy metals and oils(Sant’Anna et al. 2012).

These compounds are more common in municipal waste, including MBT, DBT and TBT(Kannan et al. 1995). The amount of TBT in studies is very different, probably due to the ban on its use in some parts of the world. The maximum level for TBT is set at 7 µg/kg in fish according to European standards(Authority EFS, 2004, Guérin et al., 2007). Based on the *meta*-analysis of TBT data in this systematic review, was estimated to be 182.33 ng/g.

In a study conducted in Japan, small amounts of TBT were found in food samples of marine origin, which indicates the prohibition of its use(Inoue et al. 2006).

In the Amodio-Cocchieri study, 33% of the samples were contaminated with DBT and 85% of farmed fish were infected with TBT(Amodio-Cocchieri et al. 2000). In Mattos study, Monobutyltin, Tributyltin, Dibutyltin concentration of different regions were examined among edible gastropod samples(Mattos et al. 2017). The observed amount were mentioned in the Table 1.

One of the products that can be contaminated with organotin compounds is edible oils. In an accident in China due to the consumption of contaminated oil led to hospitalization and death of some consumers. Tin compounds in packaged plastics migrate to oil(Gui-bin and Qun-fang, 2000). Following this incident, lard cooked with contaminated oils were examined for the amount of monomethyltin trichloride(MMT), Trimethyltin chloride(TMT) dimethyltin dichloride(DMT). Significant amounts of DMT were identified(Gui-bin and Qun-fang, 2000). Tin compounds including triphenyltin (TPhT), tributyltin (TBT), dibutyltin (DBT), di-*n*-octyltin (DOT) in various edible oils were investigated. DPhT and TPhT was non detectable (ND) in all oil samples. TBT and DBT were separated in the most of the samples(Liu et al. 2016). These two compounds were probably used as stabilizers in plastics. It is mentioned in the texts that DBT and MBT are mostly used in plastics(Liu et al. 2016). However, in this study, in addition to DBT, TBT was also isolated (Table 1). Considering that it is used as a stabilizer inside plastics, products that have plastic packaging should also be evaluated.

In Chung 's study, which measured the amount of organotin compounds in fish, the amount of TPhT was higher than other tin compounds(Chung et al. 2020). This usually depends on the water pollution in the area. In general, phenyltins is more abundant than other tin compounds. Furthermore, In the Chen study, in which the amount of organotin was measured in seafood, as in most studies, TPhT were higher in concentration than other organotin compounds(Chen et al. 2019). This is probably due to the fact that the cumulative power of TPhT is higher than TBT in food(Choi et al. 2012). The value of tolerance daily intake(TDI) for TPhT is also approved more than other compounds. This value is 0.5 μg/ kg BW for TPhT and 0.25 μg/ kg BW for TBT(Chen et al. 2019).

The data table shows that about 30% of the studies were conducted in China. Previous studies have also indicated that there is a high level of contamination of organotin in this country(Zhou et al. 2002).Two of the studies belonged to Europe. In both studies, the range of organotin was almost the same(Amodio-Cocchieri et al., 2000, Borghi and Porte, 2002). In both studies, the study was on seafood. These results re-emphasize the role of water pollution on food of marine origin.

The analysis methods included 8 gas chromatographs and one liquid chromatography (Table 1). According to previous studies, gas chromatography is commonly used(Ikonomou et al., 2002, Morabito et al., 2000, Mzoughi et al., 2005).

In this systematic review, the amount of organotin in food was investigated. Exposure to tin compounds is not limited to food. Occupational exposure also occurs(Hoppe, 2002, Ichihara et al., 2018). Therefore, determining the amount of these compounds in the blood of individuals and biomonitoring is recommended for future studies.

## Conclusion

In this systematic review, the type of organotin compounds in food was identified. Most research has been found in countries bordering the seas. Seafood showed higher levels of these compounds. so, we need more assess of seafood. According to the polished manuscript, TBT was reported more frequently than other organotin but the amount of phenyltins were higher than other compounds. The analytical methods were GC-PFPD. According to the estimate of the overall mean TBT, the amount of this type of organotin was higher than the allowable level announced by the European authorities. Due to the fact that water can be contaminated with these compounds through municipal waste, it is necessary to treat them well. Most of the reported samples in food are seafood, so sea pollution should be given more importance. One of the limitations of this systematic review study was limited to Europe, south of America and Asia and did not cover other continents. Due to the fact that these compounds are also used in pesticide, so extensive studies such as seafood have not been performed in plant products.

## Declaration of Competing Interest

The authors declare that they have no known competing financial interests or personal relationships that could have appeared to influence the work reported in this paper.
